# Multi-Platform Metabolomics Analyses Revealed the Complexity of Serum Metabolites in LPS-Induced Neuroinflammed Rats Treated with *Clinacanthus nutans* Aqueous Extract

**DOI:** 10.3389/fphar.2021.629561

**Published:** 2021-06-09

**Authors:** Amalina Ahmad Azam, Intan Safinar Ismail, Mohd Farooq Shaikh, Faridah Abas, Khozirah Shaari

**Affiliations:** ^1^ Laboratory of Natural Products, Institute of Bioscience, Universiti Putra Malaysia, Serdang, Malaysia; ^2^ Neuropharmacology Research Laboratory, Jeffrey Cheah, School of Medicine and Health Sciences, Monash University Malaysia, Subang Jaya, Malaysia

**Keywords:** neuroinflammation, clinacanthus nutans (burm. f.) lindau, serum, metabolomics, NMR, LCMS

## Abstract

The use of metabolomics as a comprehensive tool in the analysis of metabolic profiles in disease progression and therapeutic intervention is rapidly advancing. Yet, a single analytical platform could not be applied to cover the entire spectrum of a biological sample’s metabolome. In the present paper, multi-platform metabolomics approaches were explored to determine the diverse rat sera metabolites extracted from intracerebroventricular lipopolysaccharides (LPS)-induced neuroinflammed rats treated with oral therapeutic interventions of positive drug (dextromethorphan, 5 mg/kg BW); with Clinacanthus nutans (CN) aqueous extract (CNE, 500 mg/kg BW); and with phosphate buffer saline (PBS) as the control group for 14 days. Analyzed by nuclear magnetic resonance (NMR) and liquid chromatography-mass spectrometry (LC-MS) techniques, this study depicted the potential of metabolites associated with neuroinflammation and verified by MetDisease. The key observations in the perturbed metabolic pathways that showed ameliorative effects were linked to the class of amino acid and peptide metabolism involving valine, leucine, and isoleucine biosynthesis; phenylalanine, tyrosine, and tryptophan biosynthesis; and phenylalanine metabolism. Lipid metabolism of arachidonic acid metabolism, glycerophospholipid metabolism, terpenoid backbone biosynthesis, and glycosphingolipid metabolism were also affected. Current findings suggested that the putative biomarkers, especially lysophosphatidic acid (LPA) and 5-diphosphomevalonic acid from glycerophospholipid and squalene/terpenoid and cholesterol biosynthesis, respectively, showed the ameliorative effects of the drug and CN treatments by controlling cell differentiation and proliferation. Our study proved that the complex and dynamic sera profiling affected during the CN treatment was greatly influenced by the analytical platform selection as integration between the two data yielded a more holistic summary of the metabolite pattern changes. Hence, an evidence-based herb, such as CN, can be used for novel diagnostic tools in the quest for ethnopharmacological studies.

## Introduction

Neuroinflammation is a medical phase that corresponds with the protective host feedback of prolonged complex responses in any aspect of brain injury, which results in activation of glial cells, release of inflammatory mediators, such as cytokines and chemokines, and reactive oxygen and nitrogen species (Sochocka et al., 2017). When it is not treated, it could be one of the main originators of severe neurodegenerative diseases. In a recent report, *Malaysian Burden of Disease and Injury Study: 2009–2014* by the Ministry of Health (MOH), neurological diseases were shown to be among the top 12 diseases that cause morbidity and mortality in Malaysia (Ministry of Health Malaysia (MOH), 2017). Hence, the exploration of neuroprotection through the pharmacological application of anti-inflammatory agents using either synthetic drugs or natural products is rapidly increasing in popularity since both are well documented (Shal et al., 2018).

The limitation of the effective diagnostic methods for the pharmacotherapeutic intervention prompts the need for metabolomics applications. Using cells, tissues, and biofluids as the matrix in metabolomics offers a wealth of information for the metabolic profiling of direct diagnosis, therapeutic strategies, and system biology studies (Benaki and Mikros, 2018). For the targeted responses to pathogenesis, tissue metabolomics is deemed to be the most powerful platform as it provides direct information on the metabolic modification and upstream regulation (Johnson et al., 2016). However, obtaining brain samples is invasive, limiting the clinical application. Thus, serum metabolomics is a reliable choice for biomarker discovery in neuroinflammation.

*Clinacanthus nutans* (CN) extracts (CNE) are well known for their anti-inflammatory activities (Pongphasuk et al., 2005; Khoo et al., 2018). Extensive studies in a comprehensive metabolomics setting *via* proton nuclear magnetic resonance (^1^H NMR) analysis have successfully elucidated the ameliorative effects of CNE treatments in the serum (Ahmad Azam et al., 2019) and brain (Ahmad Azam et al., 2020a) of lipopolysaccharide (LPS)-induced neuroinflammed rats. The use of integrative omics between metabolomics and cytokines microarray (genomics) on brain tissue has resulted in the proposed ameliorating effects of CN treatment in an LPS-induced rat model. This study has successfully observed the increase of anti-inflammatory cytokine levels of IL2 and four and the decrease in notable biomarkers for neuroinflammation, such as choline, glutamate, and acetate, similar to the positive drug, dextromethorphan (DXM) (Ahmad Azam et al., 2020b). A metabolomics approach was also used for the serum profiling of the same CN intervention model wherein the profile of the overall mechanism has revealed several improvements in metabolic pathway of the neuroinflammation biomarkers: lactate, glucose, and pyruvate (glycolysis and gluconeogenesis); citrate and succinate (histidine); creatine, ethanol, choline, and acetate (lipid metabolism); citrate, and succinate (TCA cycle); isoleucine, leucine, and glutamate (amino acid metabolism); and 2- and 3-hydroxybutyrate (fructose and mannose metabolism, and butanoate metabolism) (Ahmad Azam et al., 2019).Yet, recently, the metabolomics community realized that a single analytical platform could not detect all metabolites in a biological sample. As a consequence, data integration between multiple analytical platforms to study a mammalian system was recommended (Dunn et al., 2011). For example, an epidemiology study in the human serum metabolome (HUSERMET) project has integrated data from gas chromatography-mass spectrometry (GC-MS), ultra-performance liquid chromatography-mass spectrometry (UPLC-MS), and nuclear magnetic resonance (NMR) spectroscopy on human serum (http://www.husermet.org/).

Combining two most widely used analytical tools, NMR and liquid chromatography-mass spectrometry (LC-MS) (Burgess et al., 2014) for metabolomics studies would enhance the NMR limitation in the analysis of a large number of low abundance metabolites and result in increased secondary metabolite detection and identification (Zhang et al., 2014). The emerging separation mode in the liquid chromatographic part uses a hydrophilic interaction column (HILIC) as the stationary phase with high organic solvent as the mobile phase in order to retain polar/ionic metabolites. In turn, this offers compatibility with the mass spectrometry (MS), making HILIC an attractive complementary tool in metabolomics studies compared to the widely used reverse-phase (RP) chromatographic columns (Theodoridis et al., 2013).

Hence, the primary objective of this study was to identify the ideal biomarkers in the serum metabolome of neuroinflammed rat model treated with CNE based on a multi-platform (NMR and LC-MS) metabolomics approach.

## Materials and Methods

### Solvents and Chemicals

All rats consumed the normal rat chow from Specialty Feeds (Glen Forrest, Australia). The phosphate buffer saline (PBS), lipopolysaccharide (LPS) derived from *Escherichia coli* 026: B6, and dextromethorphan hydrobromide (DXM) were obtained from Sigma Aldrich (St. Louis, United States). The NMR analysis used solvents in deuterated form, including deuterium oxide (D_2_O, 99.9%), deuterated methanol (CD_3_OD, 99.9%), deuterated sodium hydroxide and potassium dihydrogen phosphate were purchased from Merck (Darmstadt, Germany), and 3-Trimethylsilyl propionic acid (TSP) was purchased from Sigma Aldrich (St. Louis, United States). The LCMS analysis acquired LC-MS grade methanol, acetonitrile, water, and 0.1% formic acid from Merck (Darmstadt, Germany).

### Preparation of the CN Aqueous Extract and Phytochemical Analysis

The procedures followed the methods described by Ahmad Azam et al. (2020b). In brief, the CN plant was authenticated by a botanist and the voucher specimen (SK2883/15) was kept at the herbarium of the Institute of Bioscience, Universiti Putra Malaysia. The CN leaves collected in December 2015 at Sendayan, Negeri Sembilan, Malaysia (coordinates: 2°38'03.4"N 101°53'20.5"E) was extracted with ionized water by immersing it for 3 days at a ratio of 1 g dried leaves: 50 ml solvent. The process was repeated two times, and the extract was lyophilized (extraction yield: 30%w/w) and kept frozen in −80°C. The relative quantification of chemical markers for the CN aqueous extract *via* ^1^H NMR metabolomics approach was conducted as reported by Ahmad Azam et al. (2020b).

### Experimental Design of Neuroinflammation Rat Model

The animal tests were conducted, handled, and performed following the ethical guidelines approved by Universiti Putra Malaysia Animal Ethics Committee (Approval number: UPM/IACUC/AUP189 R070/2015). The animal experiments were carried out following the housing specification of Animal Biosafety Level-2 (ABSL-2) at Laboratory of Animal Resources, Universiti Kebangsaan Malaysia (Bangi, Malaysia) with the room temperature maintained at 24 ± 2°C, a light cycle of both dark and light for 12/12 h, and free access to food and ad libitum water.

All rats were brought in from the in-house service of the same laboratory and were acclimatized for 7 days before the experiment. Only 12 male Sprague Dawley (SD) rats of 13 weeks of age (300 ± 50 g) from the overall 35 rats published in Ahmad Azam et al. (2019) were used in this study. All rats, three in each group, were divided into the following: Group 1 was normal rats injected with phosphate-buffered saline (PBS)+water as the control group (N); Group 2 was LPS-induced rats administered with water (LPS + water); Group 3 was LPS-induced rats treated with 500 mg/kg of BW CNE (LPS+500CN); and Group 4 was LPS-induced rats treated with dextromethorphan 5 mg/kg of BW (LPS + DXM).

The induction of either 10 μl phosphate buffer saline (PBS) to the normal rat groups or lipopolysaccharides (LPS, 1 μg/1 μl) to the neuroinflammed groups have been described in previously published manuscripts (Ahmad Azam et al., 2019; Ahmad Azam et al., 2020a). To summarize, the rats were anesthetized with ketamine-xylazine (K-X); K: 80 mg/kg BW; X: 10 mg/kg of BW through intraperitoneal (i.p) route and they underwent stereotaxic surgery with a single intracerebroventricular (ICV) injection at the location of substantia nigra at the right side of the brain with a consistent rate of 3 μl per minute using a Harvard Apparatus Pump 11 elite infusion syringe *via* a Hamilton syringe (Holliston, Massachusetts, United States). In total, 1 week after the injection, the rats were administered with each treatment by oral gavage for 14 consecutive days. The CNE was prepared 3 days before each use and preserved at 4°C whereas DXM was freshly prepared before each use.

### Serum Collection

All rats from the groups were fasted for 14 h and then euthanized with K-X. Then, the terminal process *via* exsanguination was done by cardiac puncture. The serum was procured from the collected blood sample in a plain vacutainer, and centrifuged for 10 min at 4°C. The collected supernatant was stored at −80°C until analysis.

### Metabolite Fingerprinting of Serum Using ^1^H NMR Analysis

The ^1^H NMR spectroscopic serum data obtained as described in a previously published report was further utilized in this study. This was due to the success of the OPLS–DA model that revealed the potential ameliorative effects of CNE by four selected groups of normal (N), LPS + water, LPS+500CN, and LPS + DXM rats. Three representatives were randomly picked from each of the selected groups in the OPLS–DA data analysis of sera samples (Ahmad Azam et al., 2019).

### Acquisition and Pre-process of LC-MS Data of Sera

A total of 12 serum samples (3 samples from each four selected groups of N, LPS + water, LPS+500CN, and LPS + DXM) were used. Each of the 50 µl serum samples were added with 400 µl methanol in a sample tube placed on ice. The samples were vortexed for 2 min, centrifuged at 5000 rpm for 4 min at 4^o^C before 400 µl of the supernatant was transferred, and freeze-dried for 8 h. The samples were kept in −80°C for not more than 2 weeks until analyzed.

The freeze-dried samples were reconstituted with 200 µl acetonitrile: water (95:5) prior to usage. The samples were vortexed for 2 min, centrifuged for 5 min at 1,000 rpm, and then syringe-filtered using 0.45 µm, 13 mm, and PTFE filter membranes. The samples were transferred into autosampler vials from which 20 µl of the sample was injected into the Agilent 1,290 Infinity LC system coupled to Agilent 6,550 Accurate-Mass Quadrupole Time-of-Flight (Q-TOF) mass spectrometer (Agilent Technologies, United States). The HILIC column (2.1 × 100 mm, 1.7 µm, Waters, Milford, MA) was eluted using an isocratic solution of MeOH with 0.1% formic acid as the mobile phase at a constant flow rate of 300 µl/min for 15 min including the equilibration time. This method was modified from the procedure for UPLC-TOF MS on HILIC column in a broad-spectrum analysis [Center Specific Procedure (CSP) no: RTI-RCMRC-LCMS-01 ver.00] by the NIH Eastern Regional Comprehensive Metabolomics Resource Core at the Research Triangle Institute International, 2011 United States (www.rti.org/rcmrc, accessed on April 10, 2017).

An electrospray ionization (ESI) source interface was used and set in dual-mode of positive and negative. The following parameters were employed: capillary voltage, 3.5 kV; drying gas flow, 14 L/min; gas temperature: 200^o^C; nebulizer pressure, 35 psig.; fragmentor voltage, 175 V; and skimmer voltage, 65 V. The data were collected in a centroid mode, and the mass range was set at m/z 50–1,000 using the extended dynamic range. The trace of metabolites was referred to several main available computational tools for LC-MS data, namely, Agilent Masshunter Qualitative Analysis Ver. B.050.00 (MQA, RRID:SCR_019081) and Agilent Mass Profiler Professional B.05.01 (MPP), and open-source softwares, such as MZmine (RID:SCR_012040) (Pluskal et al., 2010), XCMS (RRID:SCR_015538) (Smith et al., 2016), and Metabolite Automatic Identification Toolkit (MAIT) of R package (RRID:SCR_001905) (Fernández-Albert et al., 2014). Lastly, each of the metabolite's mass representative was matched and annotated *via* MPP, HMDB MS search (HMDB, RRID:SCR_007712), KEGG (RRID:SCR_012773) compounds, Chemspider (RRID:SCR_006360) and MassBank (RRID:SCR_015535).

All processes were conducted in R wherein the LC-MS spectral baselines were corrected, normalized, aligned, and grouped by isotope. The data were binned into integrated regions with a width of 0.6934 corresponding to the aligned point. The processed data were then used for multivariate pattern recognition analysis.

### Multi-Platform Statistical Analysis

#### Metabolomics Multivariate Data Analysis (MVDA)

The preprocessed data of the different platforms were auto-scaled in the UV scale by default (van Den Berg et al., 2006). The visualization was computed for models of principal component analysis (PCA) and partial least square discriminant analysis (PLS-DA). All of the multivariate data analyses were performed using the software package SIMCA-P (version 13.0. Umetrics, Umeå, Sweden, RRID:SCR_014688). The score plots were based on the two principal components (PC1 and PC2) in which the corresponding loading plots indicated the metabolites associated with the group separation. The validation of the model was done by using the *R* ^2^ and Q^2^ values of cross-validation, CV-ANOVA, misclassification Fisher probability, and permutation test (Eriksson et al., 2006). Hierarchical cluster analysis (HCA), using Euclidean distance and Ward’s linkage method, was applied to depict the similarities of the samples analyzed by the different analytical platforms. The heat map generation was done using Metaboanalyst 3.0 (RRID:SCR_015539).

#### Metabolomics Univariate Data Analysis (UVDA)

Metabolite concentration difference based on the normalized binned data among the groups (N, LPS + water, LPS + CN500, and LPS + DXM) was evaluated in terms of fold change (FC), and the *p*-value was assessed using Student’s t-test in GraphPad Prism V 7.0 (GraphPad Software Inc., San Diego, United States, RRID:SCR_002798). The displayed metabolites with an increase (+) or decrease (−) in FC values and lower *p*-value (<0.05) were selected as significantly different.

### Pathway Analysis

The overall possible metabolic pathways were constructed using the Metabolic Pathway Analysis (MetPa, RRID:SCR_015539) (http://www.metaboanalyst.ca) (Xia et al., 2015), MetScape 3.1.1 (RRID:SCR_014687) of pathway filter and MetDisease plug-in application (Karnovsky et al., 2012; Duren et al., 2014) of the JAVA software Cytoscape ver. 3.7.1. (RRID:SCR_003032) (Nishida et al., 2014). The possible pathways were also annotated *via* the KEGG (https://www.genome.jp) and HMDB (http://www.hmdb.ca/metabolites) libraries through metabolites pathway search.

## Results and Discussion

A comprehensive phytochemical analysis for CNE was first reported under the CN project funded by the Malaysian Ministry of Agriculture (Khoo et al., 2018). The team revealed the quantification of four compounds, namely, schaftoside (0.65 ± 0.03 mg/g) followed by isovitexin (0.128 ± 0.007 mg/g), orientin (0.005 ± 0.00 mg/g), and isooreientin (0.004 ± 0.000 mg/g), *via* HPLC-DAD-ESI-MS/MS (Khoo et al., 2018). The result was parallel to our findings from which the highest relative intensities (height/area under peak) based on the binned ^1^H NMR spectra were schaftoside (0.2), vitexin (0.07), and orientin (0.009) (Ahmad Azam et al., 2019). Besides that, the recently published paper on CN aqueous extract phytochemical analysis related to anti-neuroinflammatory activities in LPS-induced BV2 cells has revealed that the bioactivity might depend on the synergistic effects between the reported 30 possible marker compounds. These markers, namely, schaftoside, acetate, propionate, alanine, clinacosides A–C, monoacylmonogalactosylglycerol, fructose, ascorbic acid, choline, stigmasterol*-β-*glucoside, citric acid, valine, catechin, orientin, chlorogenic acid, leucine, butyrate, cycloclinacoside A1 and A2, sucrose, vitexin, *β-*sitosterol, *β-*glucose, vanillic acid, gendarucin A, betulin, isoleucine, and a mixture of cerebrosides, were identified *via* ^1^H NMR metabolomics analysis of CNE treatment (Ahmad Azam et al., 2020b). Since both *in vitro* and *in vivo* results of the ^1^H NMR metabolomics approach have established that CNE can ameliorate neuroinflammation, studies integrating the *in vivo* sera results of ^1^H NMR data with LCMS data were further elaborated.

In total, 12 samples from four different groups of rat treatments used in this study were analyzed by ^1^H NMR, and LC-MS (ESI+) and (ESI−). The three sets of preprocessed data from these two analytical methods were concatenated, resulting in a total of 3,000 binned data of LC-MS (ESI+ and ESI− of each 1,375 m/z-rt bins) and 1H NMR (250 relative intensity bins). All of the data were subjected as variables in the multivariate data analysis techniques, including PCA, PLS-DA, and HCA.

The XCMS package in R was used to visualize and preprocess the LCMS data by extracting relevant information from the raw LCMS data by peak identification, matching, and nonlinear retention time correction (alignment) (Grace and Hudson, 2016). After grouping matched peaks, XCMS used the groups to identify and drift the alignment of retention time from run to run. The representatives of the aligned chromatogram using an R package and MS spectra in ESI+ and ESI− (LC-MS) for all rat serum samples obtained from all groups are provided in Supplementary Figure S1. Supplementary Figure S2 visualizes all the centroid-raw data of LCMS spectra using MQA. The sera ^1^H NMR spectra for the four groups can be referred in the published manuscript (Ahmad Azam et al., 2019).

For the multivariate data analysis, unit variance (UV) scale selection was used for both ESI+ and ESI− of the LC-MS data. To standardize all spectroscopic data, the previous Pareto ^1^H NMR sera results for the four groups were also explored using the UV scale. The use of a UV scale for the multi-platform analysis is well established and preferred as it gives equal influential importance to a model (Eriksson et al., 2006; Mumm et al., 2016). The findings from the different analytical platforms were separately visualized in the Supplementary Figure S3A–F. Almost all of the PCA and PLS-DA score plots showed a similar pattern distribution between groups based on the mean PC (principal component) scores trajectories calculated from PC-1 and PC-2.

For Supplementary Figure S3, all of the score plots for both unsupervised (PCA) and supervised (PLS-DA) models followed the rule of thumbs whereby the *R* ^2^ value was larger than Q^2^ value. A Q^2^ value greater than 0.5 is considered as the minimum requirement for appropriate use in a metabolomics analysis, yet the model is still valid if the Q^2^ is above 0 (Eriksson et al., 2006). Hence, all of the method platforms produced valid models as they complied with the *R* ^2^ and Q^2^ criteria. Each analytical platform revealed clear discrimination between normal and LPS groups treated with CN500, DXM, and water along the PC1. Although the normal and treated groups were discriminated by PC1, the distribution of each LPS-treated group with CN500 and DXM (positive drug control) could only be differentiated into two clusters by PC2. Hence, to investigate the global metabolomics alterations in the neuroinflammed rats treated with CN500 and DXM (positive control), all data acquired in both ion modes of LCMS and NMR were integrated to yield a holistic view.

The tree diagram of the HCA was utilized to observe the points that joined together sooner or later for high or low similarity, respectively (Tullis and Albert, 2013). The height of the clusters depicts the distance between each cluster using the Euclidean distance and Ward’s method. A tall vertical line means the clusters are far apart and vice versa.

### Multi-Platform Metabolomics Analysis of Sera

The PCA model for multi-platform integration data in Supplementary Figure S4 yielded an *R* ^2^ value of 0.699 and Q^2^ value of 0.495. The first PC explained 36.6% of the total variation, mainly separating the normal and LPS-induced neuroinflammed rats. Notable grouping between the groups of LPS + water, LPS + DXM, and LPS + CN500 could be observed *via* PC2, which explained 16.6% of the total variation. However, the random distribution of LPS + CN500 variables made this group difficult to be clearly discriminated. Thus, compared to the PLS-DA, the supervised method gave a clearer observation for pattern recognition, leading to the identification of the important metabolites responsible for the discrimination between groups.

### PLS-DA Analysis of the LC-MS Sera Samples

**FIGURE 1:**
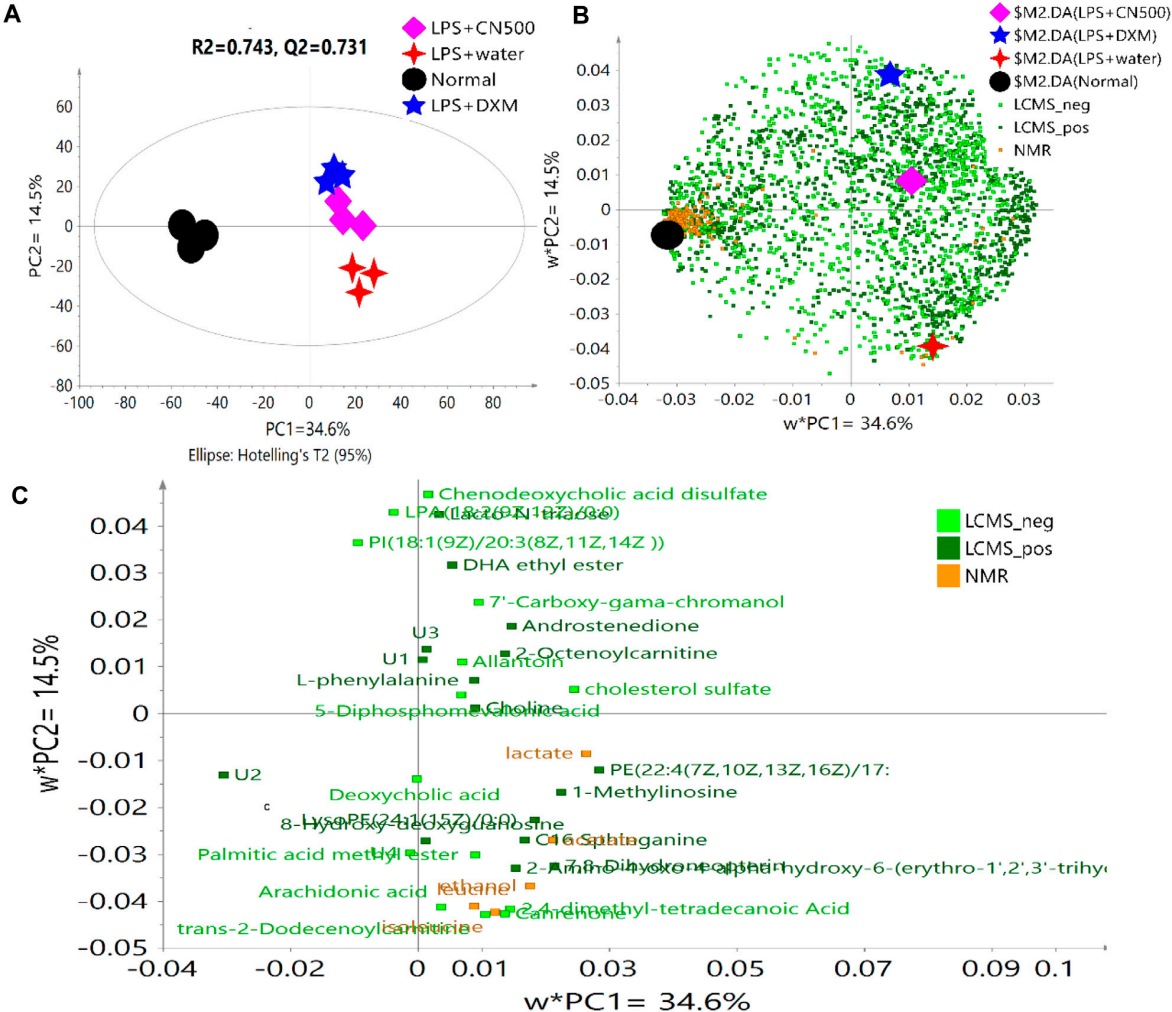
**(A)** PLS-DA score plot of four selected groups, **(B)** the overall loading scatter plot, and **(C)** loading scatter plot of the selected variables according to VIP value >1.3 of the two analytical methods. U = Unknown.

PLS-DA uses the classification technique of linear combination of original variables to classify label prediction by using zeros and ones (Westerhuis et al., 2010). Figure 1 reveals a good metabolomics model of PLS-DA with *R* ^2^ and Q^2^ values of 0.743 and 0.731, respectively, for the combination of both analytical methods of LC-MS and NMR. The variations that could be explained by each PC were 34.6% (PC1) and 14.5% (PC2) with a total of 49.1% of explainable variations in this model. As shown in Figure 1A, the score plot of PLS-DA revealed a clear discrimination along PC1 between normal (N) and LPS groups treated with DXM, CN500, and water. Rats treated with CN500 clustered together with LPS + DXM on the right side of PC1 but separated from the LPS + water group by PC2. This grouping implied the possibility of CNE positively affecting the neuroinflammatory condition as it clustered closely to the positive control (DXM).

Figure 1B reveals the complexity of the overall data compilation in the loading scatter plot. The VIP score is a weighted sum of squares of the PLS loadings, considering the amount of explained Y-variation in each dimension. The metabolites scoring VIP values higher than one were the most influential contribution to the model (Eriksson et al., 2006). However, the selection of VIP > 1 resulted in 112 putative metabolites, an amount considered as too many and difficult to be explained. Hence, the VIP indicator was increased to higher than 1.3, which appropriately categorized metabolites that yield the most influential contribution for the model discrimination. Consequently, 35 metabolites were listed as the potential biomarkers for the model (Figure 1C). The metabolites with variable importance of projection (VIP) values of 1.3 and above in the PLS-DA model are visualized in Supplementary Figure S5.

The biomarkers were determined through metabolite alteration, which was interpreted by the VIP values in MVDA. The importance of 35 putative biomarkers from three sets of analytical data is tabulated in Table 1. The application of chemometrics data, which is the mathematical statistics, from the analytical chemical analysis data is vital in a metabolomics approach (Polanski et al., 2017) that involves both the univariate (UVDA) and multivariate data analyzes (MVDA) (Pinto, 2017). When a large number of metabolites is obtained, it is natural to primarily use MVDA. Afterwards, the application of the univariate method becomes necessary to protect against the increased probability of obtaining false positives resulting from the significance testing for metabolites ranging from the tens to the hundreds by correcting multiple tests (Storey and Tibshirani, 2003; Broadhurst and Kell, 2006). The univariate method is used when only one variable is analyzed at a time.

**TABLE 1:** Major biomarkers of neuroinflammatimeon induced by LPS in rats and their fold change values due to the treatments with CN or DXM.

No	VIP value	Putative annotated metabolites	Formula	Analytical method	m/z or ppm	RT (min)	Fold change		Related pathway
							CN/LPS	DXM/LPS	
1	1.704	2-Octenoylcarnitine	C_15_H_29_NO_4_	LC-MS (ESI+)	287.20965	0.91	*↑8.02* ^*^	*↑1.81*	Lipid metabolism pathway^a^
2	1.692	Androstenedione	C_19_H_30_O_2_	LC-MS (ESI+)	290.22458	0.93	↑7.34^*^	↑2.63^*^	Steroid hormone biosynthesis^b^
									Androgen and estrogen biosynthesis and metabolism^a^ ^,^ ^c^
3	1.662	8-Hydroxy-deoxyguanosine	C_10_H_13_N_5_O_5_	LC-MS (ESI+)	283.09166	0.76	↑8.37	↑1.82	Purine metabolism^a^
4	1.658	Choline	C_5_H_14_NO	LC-MS (ESI+)	104.10753	1.16	**↑1.39**	**↓0.93**	Glycine, serine and threonine metabolism^a^ ^,^ ^b^ ^,^ ^c^
									Glycerophospholipid metabolism^a^ ^,^ ^b^ ^,^ ^c^
5	1.639	Allantoin	C_4_H_6_N_4_O_5_	LC-MS (ESI-)	158.04399	1.24	**↑1.22**	**↓0.42** ^*^	Purine metabolism^a^
6	1.619	l-Phenylalanine	C_9_H_11_NO_2_	LC-MS (ESI+)	165.07897	3.06	↑8.62	↑1.23	Phenylalanine, tyrosine and tryptophan biosynthesis^a^ ^,^ ^b^
									Aminoacyl-tRNA biosynthesis^a^ ^,^ ^b^
									Biopterin metabolism^a^ ^,^ ^c^
7	1.606	U1	C_19_H_24_N_2_	LC-MS (ESI+)	280.19394	1.02	*↑3.03* ^*^	*↑1.34*	-
8	1.576	Deoxycholic acid	C_24_H_40_O_4_	LC-MS (ESI-)	392.29265	0.84	**↑1.92**	**↓0.23**	Bile acid metabolism^*h^
									Cell signaling ^h^
9	1.558	U2	C_16_H_42_N_10_O_6_S	LC-MS (ESI+)	502.2997	1.44	↓0.94	↓0.66	-
10	1.550	U3	C_21_H_52_N_26_	LC-MS (ESI+)	668.487	2.08	↓0.49	↓0.07	-
11	1.532	Ethanol	-	NMR	1.2	-	↓0.11^**^	↓0.22^**^	Glycolysis or gluconeogenesis^a^ ^,^ ^b^ ^,^ ^c^
12	1.531	Isoleucine	-	NMR	0.92	-	↓0.42^*^	↓0.36^*^	Aminoacyl-tRNA biosynthesis^a^ ^,^ ^b^
									Valine, leucine, isoleucine biosynthesis^a^ ^,^ ^b^ ^,^ ^c^
13	1.484	Acetate	-	NMR	1.88	-	*↓0.66*	*↓0.50* ^*^	Glycolysis or gluconeogenesis^a^ ^,^ ^b^ ^,^ ^c^
									Pyruvate metabolism^a^ ^,^ ^b^
									TCA cycle^a^ ^,^ ^c^
14	1.462	7,8-Dihydropteroic acid	C_8_H_14_N_10_O_4_	LC-MS (ESI-)	314.11273	1.02	↓0.39^**^	↓0.29^**^	Folate biosynthesis^a^ ^,^ ^b^
15	1.450	Lactate	-	NMR	1.36	-	*↓0.68* ^**^	*↓0.76*	Glycolysis or gluconeogenesis^a^ ^,^ ^b^ ^,^ ^c^
									Pyruvate metabolism^a^ ^,^ ^b^
16	1.429	5-Diphosphomevalonic acid	C_6_H_14_O_10_P_2_	LC-MS (ESI-)	308.00621	0.73	**↑ 2.84**	**↓0.88**	Terpenoid backbone biosynthesis^a^ ^,^ ^b^ ^,^ ^c^
									Squalene and cholesterol biosynthesis^c^
17	1.401	Chenodeoxycholic acid disulfate	C_24_H_40_O_10_S_2_	LC-MS (ESI-)	552.20628	0.84	↑ 1.65^*^	↑ 2.03^*^	Lipid metabolism^a^
									Cell signaling^a^
18	1.400	LPA(18:2(9Z,12Z)/0:0)	C_21_H_39_O_7_P	LC-MS (ESI-)	434.24334	2.49	↑ 1.55^*^	↑ 2.59^*^	Glycerophospholipid metabolism^a^ ^,^ ^b^ ^,^ ^c^
19	1.392	Canrenone	C_22_H_28_O_3_	LC-MS (ESI-)	340.20384	0.92	↓0.52^*^	↓0.38^**^	Lipid metabolism ^a^
									Cell signaling^a^
20	1.384	Palmitic acid methyl ester	C_17_H_34_O_2_	LC-MS (ESI-)	270.25588	2.67	↑ 1.56	↑ 5.48	Glycerophospholipid metabolism^a^ ^,^ ^b^ ^,^ ^c^
21	1.383	PI(18:1(9Z)/20:3(8Z,11Z,14Z ))	C_47_H_83_O_13_P	LC-MS (ESI-)	886.55712	0.90	*↑ 1.20*	*↑ 2.12* ^*^	Cell signal ^a^
									Signal transduction ^a^
									Phospholipid metabolism ^a^
									Phosphatidylinositol phosphate metabolism^b^
22	1.372	PE(22:4(7Z,10Z,13Z,16Z)/19:O)	C_46_H_84_NO_8_P	LC-MS (ESI+)	849.62475	1.76	↓0.73	↓0.63	Glycerophospholipid metabolism^a^ ^,^ ^b^ ^,^ ^c^
23	1.367	*Trans*-2-dodecenoylcarnitine	C_19_H_35_NO_4_	LC-MS (ESI-)	341.25660	1.01	↓0.45^*^	↓0.30^*^	Lipid metabolism pathway ^a^
									Cell signaling ^a^
24	1.365	7′-carboxy-gamma-chromanol	C_20_H_30_O_4_	LC-MS (ESI-)	334.21440	0.95	↑ 2.28	↑ 1.51	Dehydrogenation carboxylate product^a^
25	1.363	1-Methylinosine	C_11_H_14_N_4_O_5_	LC-MS (ESI+)	282.09641	1.00	**↑ 1.21**	**↓0.37** ^*^	Purine metabolism^a^
26	1.340	DHA ethyl ester	C_24_H_36_O_2_	LC-MS (ESI+)	356.27153	2.13	*↑ 0.01*	*↑ 8.9* ^**^	Arachidonic acid metabolism^a^ ^,^ ^b^ ^,^ ^c^
									Biosynthesis of unsaturated fatty acids^a^ ^,^ ^b^
27	1.339	Leucine	-	NMR	0.96	-	↓0.58^*^	↓0.50^*^	Aminoacyl-tRNA biosynthesis^a^ ^,^ ^b^
									Valine, leucine, isoleucine biosynthesis^a^ ^,^ ^b^ ^,^ ^c^
28	1.337	Cholesterol sulfate	C_27_H_46_O_4_S	LC-MS (ESI-)	466.31168	0.84	**↑ 1.08**	**↓0.98**	Steroid hormone biosynthesis^a^ ^,^ ^b^ ^,^ ^c^
29	1.332	Lacto-*N*-triose	C_18_H_46_N_10_O_7_S	LC-MS (ESI+)	545.19558	1.03	↑ 2.54^*^	↑ 2.91^*^	Oligosaccharides
30	1.328	2,4-Dimethyl-tetradecanoic acid	C_16_H_32_O_2_	LC-MS (ESI-)	256.24023	1.12	↓0.53*	↓0.15^*^	Lipid metabolism ^a^
									Cell signaling ^a^
31	1.319	U4	C_19_H_17_NOS	LC-MS (ESI-)	307.10308	0.97	**↑ 1.04**	**↓0.31** ^**^	-
32	1.316	LysoPE(24:0/0:0)	C_29_H_60_NO_7_P	LC-MS (ESI-)	565.41073	1.15	**↑ 0.60**	**↓0.38**	Glycerophospholipid metabolism^a^ ^,^ ^b^ ^,^ ^c^
33	1.313	Arachidonic acid	C_20_H_32_O_2_	LC-MS (ESI-)	304.24023	2.39	*↓0.79*	*↓0.36* ^*^	Arachidonic acid metabolism ^a^ ^,^ ^b^ ^,^ ^c^
									Leukotriene metabolism ^c^
									Omega-6-fatty acid metabolism ^c^
									Prostaglandin formation from arachidonate ^c^
34	1.311	2-Amino-4-oxo-4-alpha-hydroxy-6-(erythro-1′,2′,3′-trihydroxypropyl)-5,6,7,8-tetrahydroxypterin	C_9_H_15_N_5_O_8_	LC-MS (ESI+)	321.09206	0.80	↓0.32^*^	↓0.36^*^	Biopterins and derivatives ^a^ ^,^ ^c^
35	1.303	C16 Sphinganine	C_16_H_35_NO_2_	LC-MS (ESI+)	301.29807	1.61	**↑ 0.14**	**↓0.34** ^*^	Sphingolipid metabolism ^a^ ^,^ ^b^

↑ and ↓ values denote an increase and decrease, respectively. **p < 0.001, and *p < 0.05 show significant differences as compared to LPS + water (negative control). Fold change (FC) value in bold represents metabolites difference in pattern alteration between DXM and 500CN, while *italic* serves for metabolites which have either one value significant between DXM or CN500, respectively. The possible pathway was suggested by.

a Pathway from KEGG and HMDB.

b For MetaboAnalyst 3.0 pathway analysis.

c Metscape plugin in Cytoscape.

Therefore, the UVDA was conducted to obtain the metabolites alteration expressed in fold change (FC). The summary of the metabolite properties such as mass data, chemical shifts (ppm), chemical formulations, related pathways, and their FC value was listed. In Table 1, the FC displayed only focused on LPS-induced neuroinflammed rats treated with DXM or CN500. The herb (CN500) and drug (DXM) treatments yielded consistent results of amelioration without normalization. Hence, the comparison was focused only on LPS + water treatment to understand the relationship of the chemical interventions on the neuroinflammed rats. Among the 35 VIP > 1.3 metabolites, only nine of them differed in pattern alteration (fold change increase or decrease, bolded; Table 1) between the biomarker of DXM and CN500. The metabolites that increased in CN treatment were identified as choline, allantoin, deoxycholic acid, 5-diphosphomevalonic acid, 1-methylinosine, cholesterol sulfate, unknown 4 (U4), LysoPE(24:0/0:0), and C16 sphinganine. Similar significant metabolite alteration was also observed in the DXM group, suggesting that both DXM and CN treatments produced a similar pattern in ameliorating the neuroinflammed condition of LPS-induced rats. Only allantoin, 1-methylinosine, U4, and C16 sphinganine were significantly different between the treatments of CN500 and DXM.

Among the remaining 26 metabolites, only seven metabolites in either one of the group treatments (LPS + CN500 or LPS + DXM) significantly differed in intensity (FC value in italic in Table 1). The comparison between these two treatments showed a significant difference in the increase of 2-octenoylcarnitine, U1 in the CN treatment group, whereas PI(18:1(9Z)/20:3(8Z,11Z,14Z)), DHA ethyl ester, and arachidonic acid decreased in the DXM group. The significant decrease of lactate could only be observed by CN treatment. For the remaining 19 metabolites, androstenedione, chenodeoxycholic acid disulfate, LPA(18:2(9Z,12Z)/0:0), and lacto-*N*-triaose have uniformly signified increase in both groups. The significant fold change (FC) decrease for both treatments was observed in isoleucine, ethanol, 7-8-dihydropteroic acid, canrenone, *trans*-2-dodecenoylcarnitine, leucine, 2,4-dimethyl-tetradecanoic acid, and 2-amino-4-oxo-4-alpha-hydroxy-6-(erythro-1',2',3'-trihydroxypropyl)-5,6,7,8-tetrahydroxypterin. However, 8-hydroxy-deoxyguanosine, l-phenylalanine, palmitic acid methyl ester, and 7'-carboxy-gamma-chromanol equally increased without any significance. The rest of the compounds, including U2, U3, and PE(22:4(7Z,10Z,13Z,16Z)/19:O) decreased in intensity, yet these changes were not significant.

### Validation of PLS-DA of Multi-Platform Model

One of the problems mostly encountered in building PLS models in MVDA is the greediness of the technique, leading to the overfitting of the data. This can be controlled by using other appropriate cross-validation strategies (Kjeldahl and Bro 2010; Westerhuis et al., 2010), such as permutation test, CV-ANOVA test, misclassification Fisher probability and double cross-validation. Hence, the present PLS-DA of the multi-platform model was validated by the confirmation of the significant CV-ANOVA value of *p* = 0.034. The validation was further observed in the misclassification table in which the Fisher probability value obtained was 6.5e-5. The correct classification ratio was calculated to be 100%, meaning that all rats were correctly predicted by the model. Both CV-ANOVA and Fisher probability values of less than 0.05 proved the validity of the model (Eriksson et al., 2006). The CV-ANOVA and misclassification table were tabulated in Supplementary Tables S6, S7, respectively.

The internal cross-validation of the model can be referred back to *R* ^2^ and Q^2^ values whereby the model was proven to be an established metabolomic model. Unfortunately, the permutation test did not fit into the first regulations of *R* ^2^ < 0.3 and Q^2^ < 0.05 (Eriksson et al., 2008). The *R* ^2^ values of the model for each group were 0.56, 0.54, 0.57, and 0.47 while the Q^2^ values were −0.03, −0.22, −0.29, and −0.23 for normal, LPS + CN500, LPS + water, and LPS + DXM, respectively. However, there was another criterion for metabolomic model validation in which all Q^2^ values on the permuted data set to the left would be lower than the Q^2^ value of the actual data set on the right or the regression line (line joining the point of observed Q^2^ to the centroid of a cluster of permuted Q^2^) had a negative value of *Y*-intercept. Hence, this criterion for the permutation test proved the validity of the PLS-DA model as all four permuted classes yielded negative Q^2^ intercept values (Mahadevan et al., 2008; Sedghipour and Sadeghi-Bazargani, 2012). The permutation tests are shown in Supplementary Figure S8A–D in the Supplementary Material.

### Comparative Results From the Previous Study

A published study of the same project on ^1^H NMR serum has shown seven intervened biomarkers: choline, allantoin, ethanol, isoleucine, acetate, lactate, and leucine (Ahmad Azam et al., 2019). However, the use of UVDA on the quantitative results (Table 1) revealed a complexity due to the inconsistent pattern changes of each biomarker when two analytical tools were combined. Hence, the MVDA of the component analysis was referred to holistically summarize the pattern changes of both analytical observations. The PLS-DA model cluster analysis resulting in the groups of nested trees could also assist in recognizing the variations in the metabolites of LCMS and NMR data.

#### The Hierarchical Cluster Analysis (HCA) of the PLS-DA Model

**FIGURE 2:**
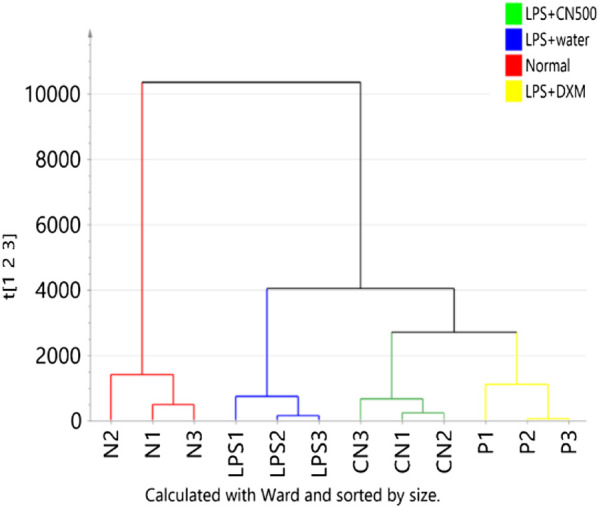
HCA of the four groups metabolome (N = normal; LPS = LPS + water; CN = LPS + CN500 and P = LPS + DXM; *n* = 3) as classified by two analytical platforms. Samples are color-coded according to the clustered classes.

The HCA of Euclidean distance and Ward’s linkage method in Figure 2 illustrated the division of the samples analyzed by the two analytical platforms. The first cluster break was between the normal *vs.* LPS-induced neuroinflammed rat groups. The LPS-induced neuroinflammed groups were then further divided into two groups: water and the CN500- and DXM-treated group. Although the CN500 treatment did not effectively regulate the metabolites up to those of the normal rats, CN500 improved the conditions of the neuroinflammed rats as the metabolites clustered together with those of the positive control, DXM. Both CN500 and DXM groups were well separated from the water-treated neuroinflammed rats, suggesting the possible ameliorative effects of these treatments on LPS-induced neuroinflammed rats.

**FIGURE 3:**
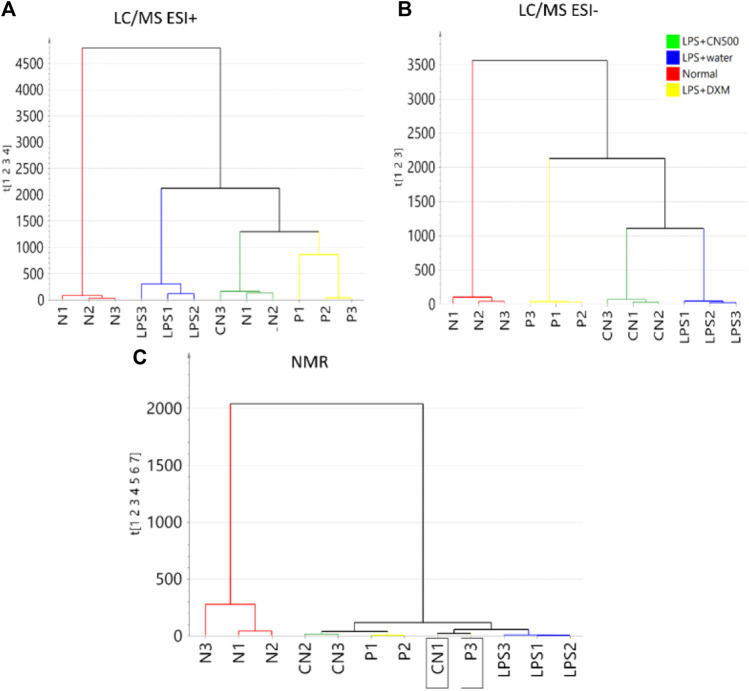
HCA of sera metabolite profiles of the four groups (*N* = normal; LPS = LPS + water; CN = LPS + CN500 and P = LPS + DXM; *n* = 3) with a separate analytical platform of **(A)** LC/MS (ESI+), **(B)** LC/MS (ESI-), and **(C)** 1H NMR. The labelled boxes indicate samples that were mixed classified.

Figures 3A–C represent the HCA dendrograms of separate analytical data of LC-MS (ESI+ and ESI−) and 1H NMR, respectively. The LC-MS (ESI+) showed a similar tree cluster to that in Figure 2 while the other two dendrograms showed a similar separation only between normal and LPS-induced neuroinflammed rats. The LC-MS (ESI-) data differed in the classification as LPS + CN500 was clustered with the LPS + water group. However, the NMR data produced an unclear HCA classification tree because the CN500 and DXM (P2) samples were mixed. Furthermore, each sample from LPS + DXM and LPS + CN500 branched together with LPS + water.

**FIGURE 4:**
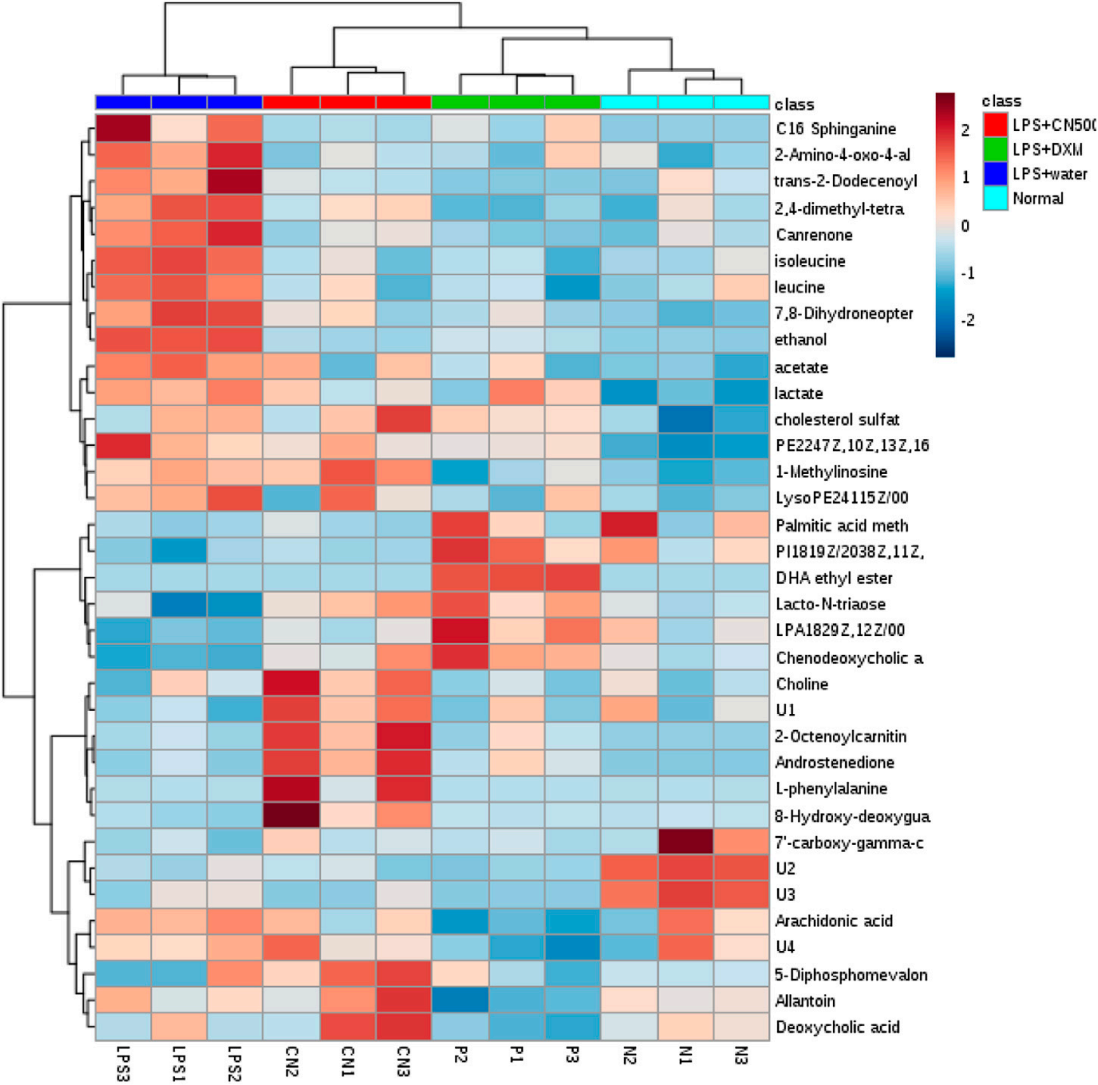
Heat map of the 37 biomarkers in normal, LPS-induced, LPS+500CN, and LPS + DXM rats sera based on HCA using Euclidean distance and Ward’s minimum variance method. The concentration of each metabolite is coloured based on a normalized scale of minimum −2 (dark blue) to a maximum of 2 (dark red).

The 35 putative biomarkers were further visualized using heatmap computed using MetaboAnalyst 3.0 (Figure 4). The heatmap successfully classified LPS + CN500, LPS + DXM, and normal rats together as a group that differed from the LPS + water group.

### Pathway Analysis

**FIGURE 5:**
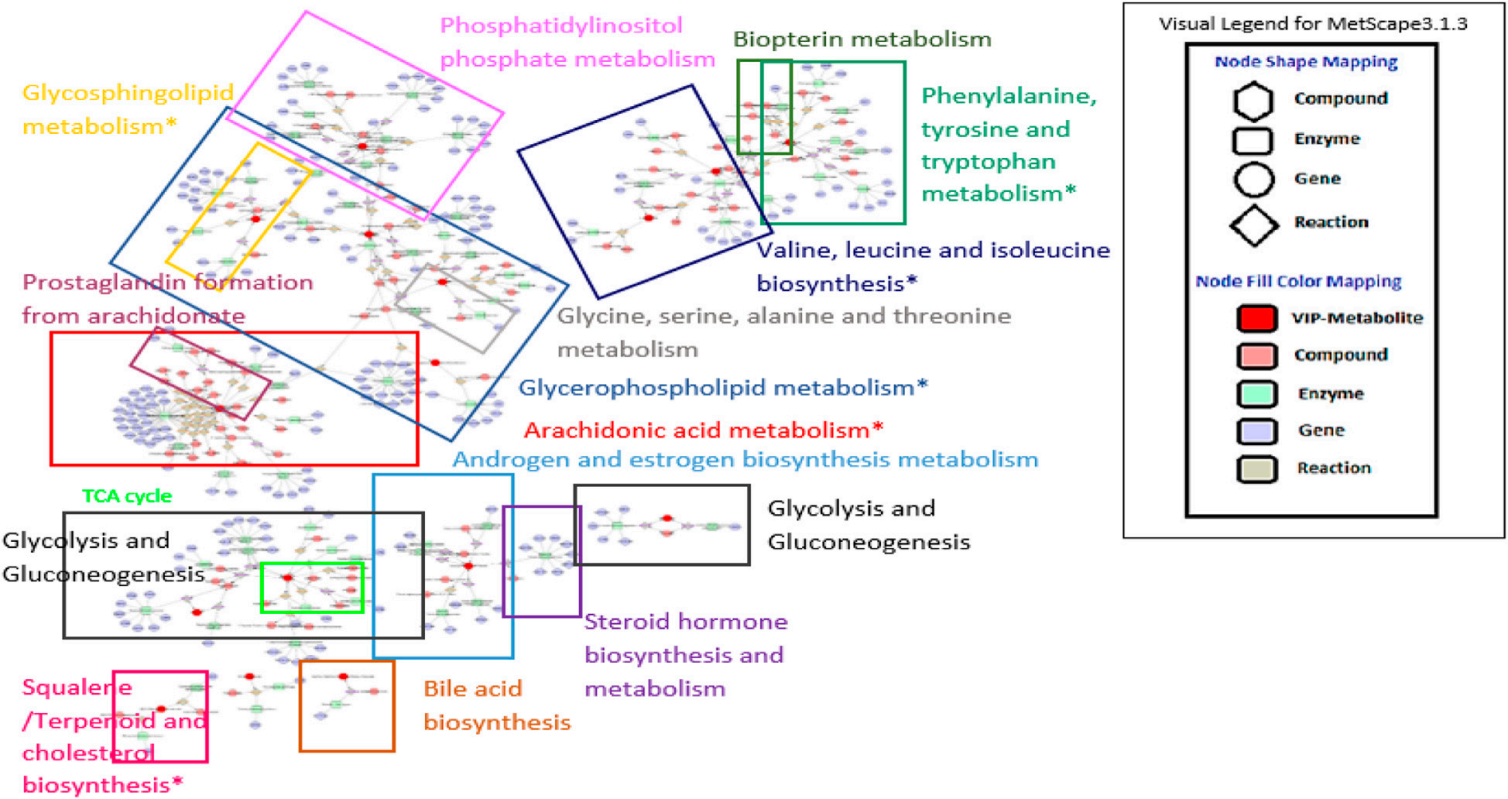
Suggested metabolic system by MetScape. The map summarizes the shortest route to explain interactions among metabolites with VIP value > 1.3. Sign * indicates the most affected pathway based on the ingenuity pathway analysis computed from MetPa analysis. The clear image can be accessed at http://www.ndexbio.org/#/network/d77dd163-9ad1-11e9-8bb4-0ac135e8bacf?accesskey=f1aa0859c5f3b7d9c5507942b02596bd3a1eee60414d960f22920a3a68c10701.

**FIGURE 6:**
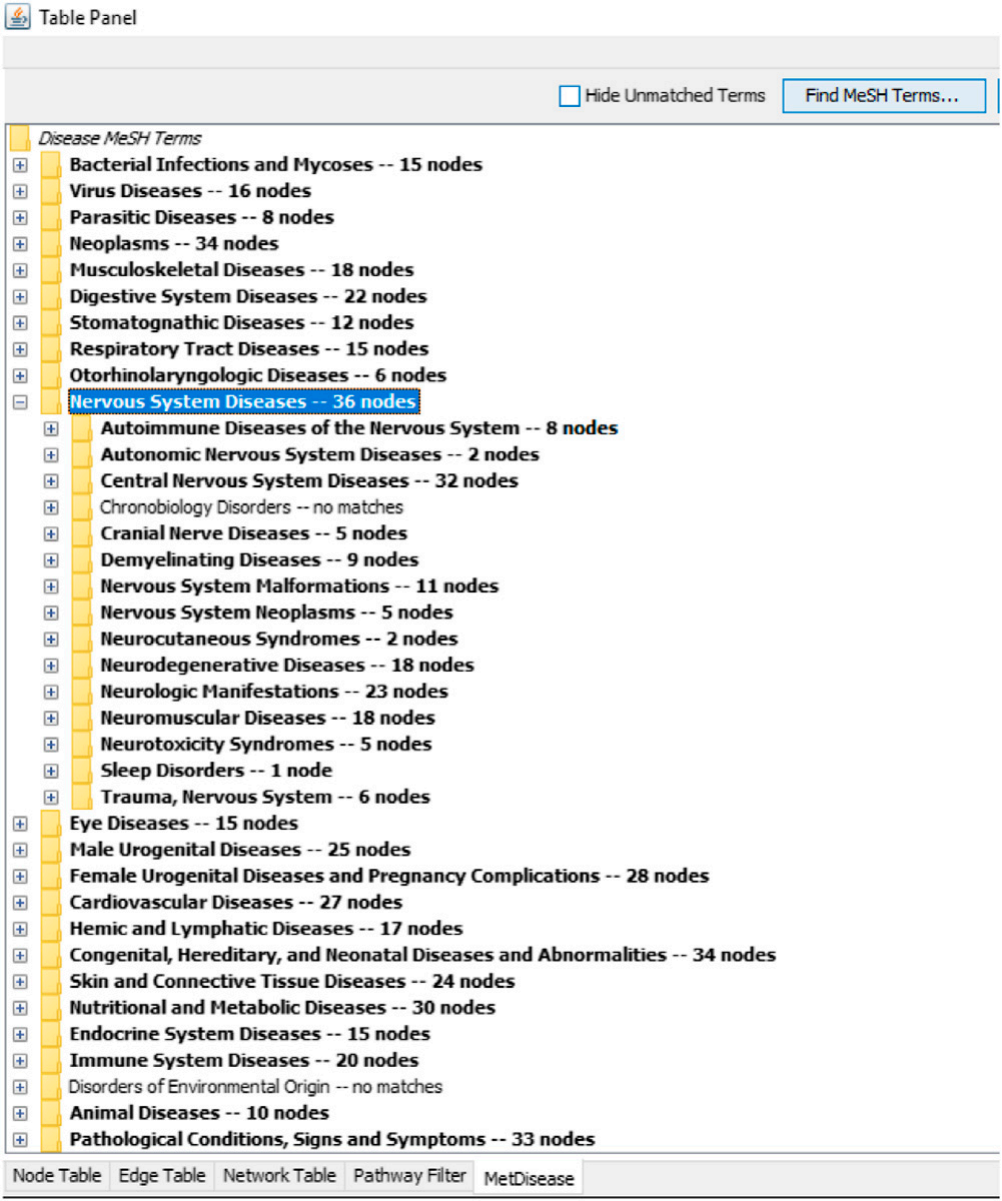
The compatibility of the 31 biomarkers with nervous system diseases was recorded to be the highest by 36 nodes *via* a successful linkage in MetDisease.

The results shown by the ingenuity metabolic pathway analysis (MetPA) index by MetaboAnalyst suggested seven most impacted pathways based on the input of 31 metabolites (four unknowns were excluded). The valine, leucine and isoleucine biosynthesis; phenylalanine, tyrosine, and tryptophan biosynthesis; phenylalanine metabolism; arachidonic acid metabolism; glycerophospholipid metabolism; terpenoid backbone biosynthesis; and sphingolipid metabolism were pathways showing an impact value higher than 0.1. This value was required to be categorized as the most relevant pathway. The summary of pathway analysis suggested by MetPA was visualized and tabulated in the Supplementary Figure S9 and Supplementary Table S10, respectively. Only two of the pathways (glycolysis and gluconeogenesis; and valine, leucine, and isoleucine biosynthesis) were in agreement with the previous ^1^H NMR sera profiling results (Ahmad Azam et al., 2019). However, all of the other suggested relevant pathways from the previous and recent reports were compatible with the overall systemic map suggested by MetScape ver. 3.1.3. Figure 5 summarizes the shortest route to explain the interactions among the metabolites. Further analysis was conducted with another plugin in Cytoscape called MetDisease. This plugin matched the metabolites to those reported in PubChem Compound record under their medical subject headings (MeSH) (Duren et al., 2014). As a result, Figure 6 is a list of the promising outcomes from MetDisease analysis from which the compatibility of the metabolites with nervous system diseases was recorded to be the highest by 36 nodes of successful linkages. Hence, the model of this study has been demonstrated as an established neuroinflammation model since the metabolites were mostly well-matched with the central nervous system diseases by 36 nodes.

Almost half of the resulted (18/31) biomarkers were categorized in the chemical taxonomy as a superclass of lipid and lipid-like molecules (Human Metabolome Database (HMDB), 2018). They were identified as 2-octenoylcarnitine, androstenedione, deoxycholic acid, chenodeoxycholic acid disulfate, LPA(18:2(9Z,12Z)/0:0), PE(22:4(7Z,10Z,13Z,16Z)/19:O), LysoPE(24:0/0:0), canrenone, arachidonic acid, DHA ethyl ester, palmitic acid methyl ester, *trans*-2-dodecenoylcarnitine, PI(18:1(9Z)/20:3(8Z,11Z,14Z )), 7'-carboxy*-γ-*chromanol, 2,4-dimethyl-tetradecanoic acid, cholesterol sulfate, choline, and C16 sphinganine. Most of these metabolites were located at the right side of the loading scatter plot (Figure 1C) which belonged to the LPS-induced rats. Lipids are a crucial member of the cellular membrane and source of stored energy metabolism, and they act as a signaling transductor at the membrane cell (van Meer et al., 2008).

The model pathway analysis results showed a substantial influence among lipid metabolisms *via* glycerophospholipid, glycosphingolipid and arachidonic acid metabolism alteration. Glycerophospholipids, such as phosphatidylethanolamine (PE) and LysoPE are important components of membrane bilayers. Lysophosphatidic acid (LPA) is extremely important in the biochemical process as a lipid mediator that controls growth, mortality, and differentiation of chemotaxis and ultrastructure of human neutrophils for the innate immune system (hmdb.ca, 2019). The separation by PC2 in Figure 1C shows the location of PE and lysoPE to be higher in LPS + water while LPA was observed among LPS + DXM, LPS + CN500, and normal rats. Consequently, the LPS induction has successfully resulted in lipid rafts on the serum lipid membranes since an amphiphilic structure of LPS allowed the lipids to be rapidly inserted into the cell membranes, forming into the disordered phase of lipid ratios (Martins, 2016). Sphingolipid is also the backbone of neural tissue. The induction of LPS has been reported to interrupt the blood-brain barrier (BBB), increasing the level of the sphingolipid C16 sphinganine in the serum of LPS + water rats (Figure 1C). Furthermore, the roles of squalene/terpenoid and cholesterol biosynthesis in the ameliorative effects of DXM and 500CN treatments were demonstrated by the putative biomarker of 5-diphosphomevalonic acid *via* the mevalonate pathway. This pathway leads to the synthesis of sterol and isoprenoids, which have been recognized to be essential for cell proliferation for the survival of various types of cancer cells (Ameer et al., 2018). The high abundance of arachidonic acid, which is a polyunsaturated and essential fatty acid, among the LPS + water rats was reported to be important in the inflammation-associated disease as it could act as a mediator to regulate leukocyte chemotaxis, inflammatory cytokines, and immune function (Thakur et al., 1998). Nevertheless, further lipids analysis needs to be confirmed with a proper lipidomic analysis.

Another class of the possible biomarkers was nucleotides and analogs (hydroxyl-deoxyguanosine and 1-methylinosine); organic acid and derivatives (phenylalanine, isoleucine, leucine, acetate, and lactate); organoheterocyclic compounds (allantoin, 7,8-dihydropteroic acid and 2,4-dimethyl-tetradecanoic acid); and organooxygen compounds (ethanol, 5-diphosphomevalonic acid and lacto-*N*-triose). As the parallel relationships between the metabolites in the pathways involved in the nervous system diseases were deciphered, we suggest looking into the signaling of neurotransmission. The strongest impact obtained from MetPa was observed on the valine, leucine and isoleucine biosynthesis, and phenylalanine, tyrosine, and tryptophan metabolism. The production of essential amino acids, such as phenylalanine and branched-chain amino acid (BCAA) (isoleucine and leucine), could influence the neurotransmitter levels in the brain. This study revealed the elevation of BCAA in the serum of LPS + water group, confirming the inflammation as an activation of the nervous system. Unfortunately, due to the lack of LCMS databases, the identification of some important metabolites in the spectra could not be completely accomplished. Thus, the overall suggested metabolic system was only based on the 31 identified biomarkers (Figure 5).

## Conclusion

The findings from both analytical data sets revealed the complexity of serum metabolites, exhibiting the success of LPS induction in producing a neuroinflammed rat model. An ameliorative effect could be suggested as a result of CN treatment. The multi-platform cross-validation models have allowed an in-depth understanding and holistic view of the metabolite variations; hence, a novel diagnostic tool was established. However, for future research, these obtained results should be validated with a larger prospective cohort by integrating other omics.

## Data Availability

The datasets presented in this study can be found in online repositories of excel workbook format at the private url for figshare: https://figshare.com/s/20717feb8de862a5a4ee as public DOI (htpps://doi.org/10.6084/m9.figshare.13089149) will be provided after the article publishes.

## Glossary

ANOVA: analysis of variance
BBB: blood-brain barrier
BW: body weight
CN: Clinacanthus nutans
CV: coefficient of variance
DHA: DXM: dextromethorphan
ESI+: positive electrospray ionization
ESI-: negative electrospray ionization
FC: fold change
HCA: Hierarchical cluster analysis
HILIC: hydrophilic interaction column
HMDB: human metabolome databse
HUSERMET: human serum metabolome
ICV: intracerebroventicular
i.p: intraperitoneal
KEGG: Kyoto encyclopedia of genes and genomes
K-X: ketamine-xylazine
LC-MS: liquid chromatography-mass spectrometry
LPA: lysophosphatidic acid
LPS: lipopolysaccharides
MAIT: metabolite automatic identification toolkit
MetPa: metabolic pathway analysis
MQA: Agilent masshunter qualitative analysis
MPP: Agilent mass profiler professional
MVDA: multivariate data analysis
NMR: nuclear magnetic resonance
OPLS-DA: orthogonal partial least squares discriminant analysis
PBS: phosphate buffer saline
PC: principal component
PCA: principal component analysis
PE: phosphatidylethanolamine
PI: phosphatidylinositol
PLS-DA: partial least square discriminant analysis
RP: reverse‐phase
SIMCA: statistical isolinear multiple component analysis
TCA: tricarboxylic acid cycle
U: unknown
UV: unit variance
UVDA: univariate data analysis.
