# Supplementary material for: Multi-Platform Metabolomics Analyses Revealed the Complexity of Serum Metabolites in LPS-Induced Neuroinflammed Rats Treated with Clinacanthus nutans Aqueous Extract
Source: Front Pharmacol. 2021 Jun 9;12:629561. doi: 10.3389/fphar.2021.629561 (PMC8220158; doi:10.3389/fphar.2021.629561)
Supplement: Supplementary file 1 [file DataSheet1.docx]

Supplementary Material


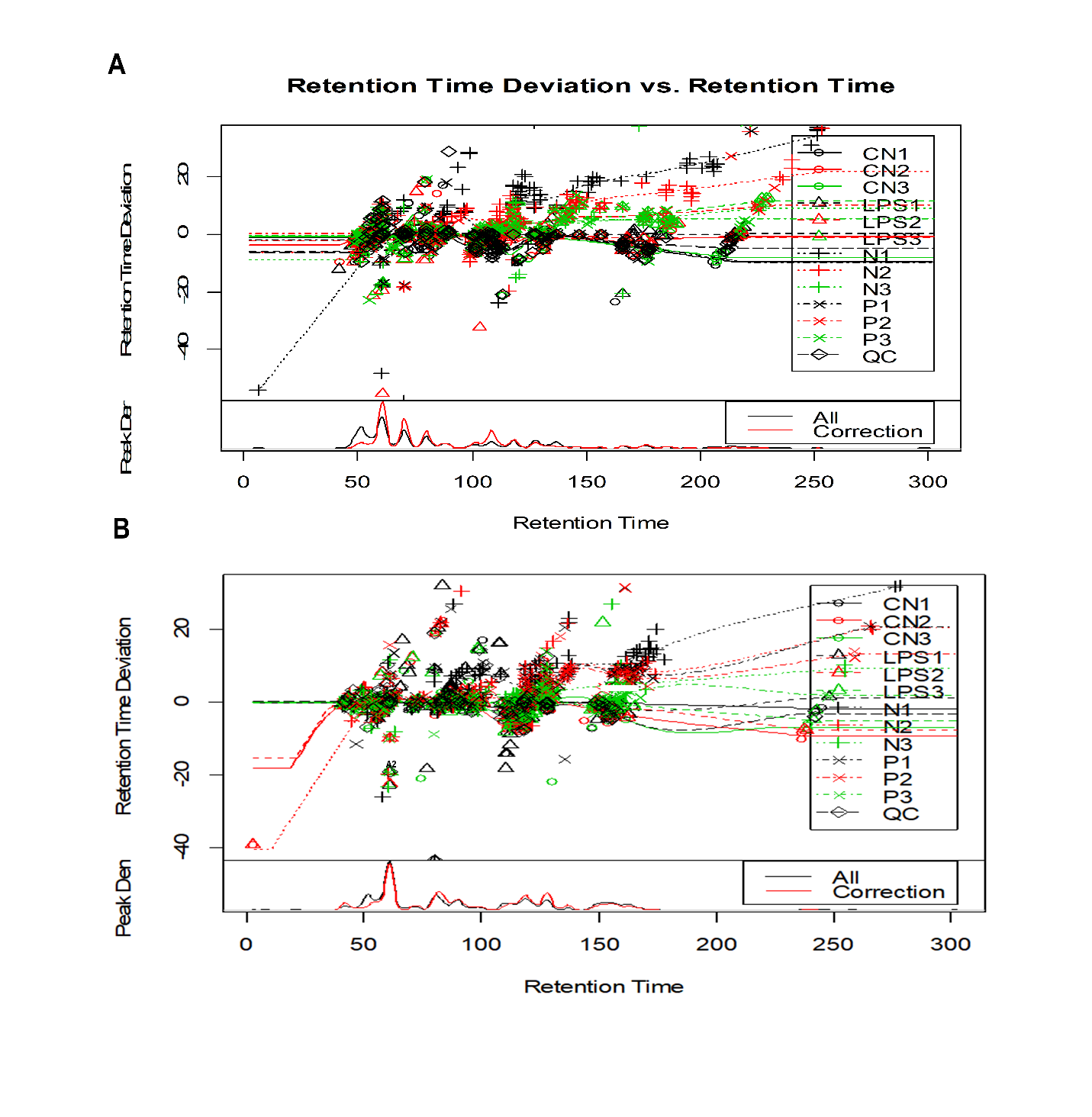


**Supplementary Figure S1.** Aligned of all preprocess chromatogram peak spectra density vs retention time (A) with positive ion and (B) negative ion of chromatogram MS-spectra. The representative of normal rat (N), LPS rat treated with dextromethorphan (P), LPS rat treated with 500mg/kg BW (CN) and LPS rat treated with water (LPS)

| **Group/Method** | **ESI (+ve)** | **ESI (-ve)** |
| --- | --- | --- |
| **Normal** |  |  |
| **LPS+water** |  |  |
| **LPS+DXM** |  |  |
| **LPS+CN500** |  |  |

**Supplementary Figure S2.** Overlaid centroid-raw spectra of Total Ion Chromatogram (TIC) vs retention time for three biological samples for each groups treatment with positive ion (ESI +ve) and negative ion (ESI –ve) of rats’ sera MS-spectra.

| ***Analytical method*** | ***PCA*** | ***PLS-DA*** |
| --- | --- | --- |
| *LC-MS*  *(ESI+)*  **C D** |   **A B** |  |
| *LC-MS*  *(ESI-)*  **E F** |  |  |
| *NMR* |  |  |

**Supplementary Figure S3.** PCA and PLS-DA score plots of LC-MS (ESI+) (A and B), LC-MS (ESI-) (C and D) and NMR (E and F) of 4 different rat treatments.


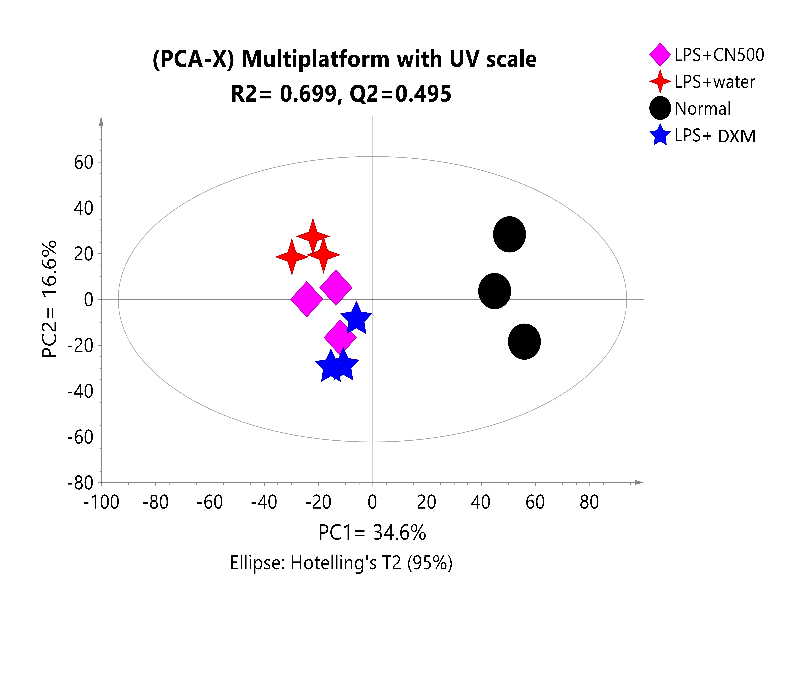


**Supplementary Figure S4.** PCA score plot of different groups treatment

**Supplementary Figure S5.** Metabolites with variable importance of projection (VIP) values ≥ 1.3 of the PLS-DA model.

**Supplementary Table S6.** CV-ANOVA table of PLS-DA of multiplatform model

|  | **SS** | **DF** | **MS** | **p** | **SD** |
| --- | --- | --- | --- | --- | --- |
| **Total corr.** | 33 | 33 | 1 | - | 1 |
| **Regression** | 22.2472 | 15 | 1.48315 | 0.0342939 | 1.21784 |
| **Residual** | 10.7528 | 18 | 0.597378 | - | 0.772903 |

SS= Sum of squares, DF= degree of freedom, MS= mean square, p=significant value, SD= standard deviation

**Supplementary Table S7.** Misclassification table of PLS-DA of multiplatform model

|  | ***Members*** | ***Correct*** | ***LPS+***  ***CN500*** | ***LPS+***  ***water*** | ***Normal*** | ***LPS+***  ***DXM*** | ***No class (YPred <= 0)*** |
| --- | --- | --- | --- | --- | --- | --- | --- |
| *LPS+CN500* | 3 | 100% | 3 | 0 | 0 | 0 | 0 |
| *LPS+water* | 3 | 100% | 0 | 3 | 0 | 0 | 0 |
| *Normal* | 3 | 100% | 0 | 0 | 3 | 0 | 0 |
| *LPS+DXM* | 3 | 100% | 0 | 0 | 0 | 3 | 0 |
| *No class* | 0 |  | 0 | 0 | 0 | 0 | 0 |
| *Total* | 12 | 100% | 3 | 3 | 3 | 3 | 0 |
| *Fisher's prob.* | 6.5e-005 |  |  |  |  |  |  |

|  |  |   **C D**  **A B** |
| --- | --- | --- |
|  |  |  |

**Supplementary Figure S8.** Permutation tests for the PLS-DA model for classes of (A) Normal, (B) LPS+CN500, (C) LPS+water and (D) LPS+DXM.


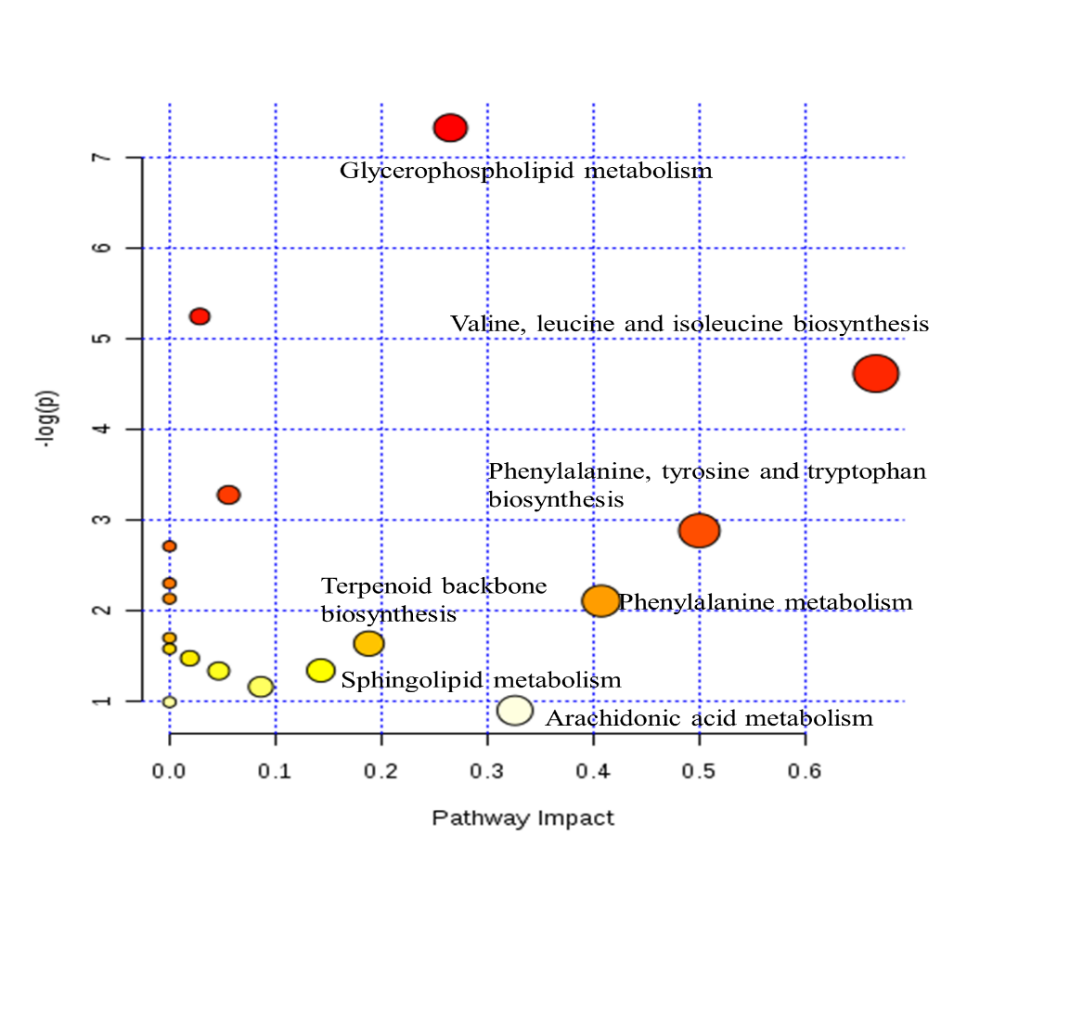


**Supplementary Figure S9.** A summary of pathway analysis by MetPA

**Supplementary Table S10.** Ingenuity pathway analysis by MetaboAnalyst (MetPA)

|  | **Metabolism** | **Total** | **Expected** | **Raw p** | **-log** | **Holm adjust** | **FDR** | **Impact** |
| --- | --- | --- | --- | --- | --- | --- | --- | --- |
| **1** | Valine, leucine and isoleucine biosynthesis | 11 | 0.157 | 0.010 | 4.620 | 0.778 | 0.27 | 0.667 |
| **2** | Phenylalanine, tyrosine and tryptophan biosynthesis | 4 | 0.057 | 0.056 | 2.884 | 1.000 | 0.90 | 0.500 |
| **3** | Phenylalanine metabolism | 9 | 0.128 | 0.122 | 2.107 | 1.000 | 1.00 | 0.407 |
| **4** | Arachidonic acid metabolism | 36 | 0.514 | 0.408 | 0.897 | 1.000 | 1.00 | 0.326 |
| **5** | Glycerophospholipid metabolism | 30 | 0.428 | 0.001 | 7.336 | 0.053 | 0.05 | 0.265 |
| **6** | Terpenoid backbone biosynthesis | 15 | 0.214 | 0.195 | 1.636 | 1.000 | 1.00 | 0.188 |
| **7** | Sphingolipid metabolism | 21 | 0.300 | 0.262 | 1.339 | 1.000 | 1.00 | 0.143 |

*FDR- False discovery rate
